# Cardiovascular Safety of Antifracture Medications in Patients With Osteoporosis: A Narrative Review of Evidence From Randomized Studies

**DOI:** 10.1002/jbm4.10522

**Published:** 2021-06-23

**Authors:** Alexander J Rodríguez, Bo Abrahamsen

**Affiliations:** ^1^ Bone and Muscle Health Research Group, Department of Medicine, School of Clinical Sciences, Faculty of Medicine, Nursing and Health Sciences Monash University, Monash Medical Centre Clayton Victoria Australia; ^2^ Disorders of Mineralisation Research Group, School of Medical and Health Sciences Edith Cowan University Joondalup Western Australia Australia; ^3^ Department of Medicine Holbæk Hospital Holbæk Denmark; ^4^ Odense Patient Data Explorative Network (OPEN) University of Southern Denmark Odense Denmark

**Keywords:** CARDIOVASCULAR, EVIDENCE‐BASED MEDICINE, MEDICATION SAFETY, OSTEOPOROSIS, RANDOMIZED CONTROLLED TRIALS

## Abstract

Osteoporosis and cardiovascular (CV) disease share common risk factors and pathophysiology. Low bone mineral density (BMD) and fractures appear to increase the risk for multiple CV diseases. Equally, prevalent CV disease appears to predispose to bone loss and increase fracture rates. This relationship has naturally provoked the hypothesis that stopping bone loss may result in some CV benefit. Secondary analyses of safety and adverse event data from many randomized controlled trials (RCTs) have attempted to clarify this putative association. Recently, the discontinuation of odanacatib (anti‐cathepsin K monoclonal antibody) over stroke concerns and the imbalance in ischemic events in romosozumab‐treated (anti‐sclerostin monoclonal antibody) women compared to bisphosphonate‐treated women, has provided further justification to better characterize potential CV benefits and harms of osteoporosis medications. This review delves into the seminal, and other major RCTs of osteoporosis medications and, using both published data and additional information provided on trial registration pages, examines the evidence for CV safety and harms of these medications. Accepted and emerging “off‐target” effects are explored for validity, biological plausibility, and clinical importance. A brief research agenda is provided to stimulate the next wave of clinical development and CV understanding of osteoporosis medications. © 2021 The Authors. *JBMR Plus* published by Wiley Periodicals LLC on behalf of American Society for Bone and Mineral Research.

## Introduction

Osteoporosis is a systemic skeletal disorder characterized by the loss of bone mass and deterioration in bone microarchitecture. These microstructural alterations combine to compromise bone strength leading to skeletal fragility and increased susceptibility of fracture.^(^ ^1^ ^)^

Fractures are a devastating outcome. Recovery from a fracture is costly, takes many months and indeed some never truly recover and lose independence. Indeed, mortality and the development of comorbid disease, greatly increases after most types of fractures but particularly hip fractures.^(^ ^2^ ^)^ Recent health economic literature suggests that fractures pose a higher disease and cost burden than most cancers (except lung cancer) but do not attract as much public attention or institutional funding.^(^ ^3^ ^)^

Osteoporosis does not exist in isolation and indeed may either precede, develop concomitantly, or present secondary to other comorbid diseases, analogous to how cardiovascular (CV) disease may develop subsequently to type 2 diabetes mellitus. The association between CV disease and osteoporosis is of profound importance because CV disease is overrepresented as a cause of death in patients who have fractured; and fractures are common in patients with known CV disease. Atherosclerosis shares many risk factors with osteoporosis and is hypothesized to establish in the decades of life when the net flux of available calcium becomes deranged; “switching” from skeleton deposition to renal filtration and deposition in vessel walls and other soft tissues.^(^ ^4^ ^)^ Certainly, on a biological level, there appears to be bidirectionality between bone and vascular disease.^(^ ^5^ ^)^ Mechanisms underlying common aging, such as chronic inflammation as well as the decline in renal function predisposing to chronic kidney disease (itself significantly effecting calcium and mineral balance) are all thought to interact in the eventual co‐manifestation of osteoporosis and CV disease (Table 1).

**Table 1:** Clinical Evidence for Bidirectionality Between Skeletal Disease and Vascular Disease

Bone → vascular	Vascular → bone
Prevalent vertebral fracture predicted cardiac events in older women → HR = 2.9 (95% CI, 1.7–4.9)^(^ ^6^ ^)^	Diagnosis of atrial fibrillation increased risk of hip fracture → HR (men) = 1.97 (95% CI, 1.61–2.52) & HR (women) = 2.08 (95% CI, 1.90–2.39)^(^ ^7^ ^)^
Whole‐body BMD loss predicted cardiac events in older men → HR = 1.78 (95% CI, 1.05–3.03)^(^ ^8^ ^)^	Stroke and MI increased the risk of hip fracture → HR (stroke) = 3.86 (95% CI, 3.25–4.59) & → HR (MI) = 1.85 (95% CI, 1.54–2.21)^(^ ^9^ ^)^
Total hip BMD predicted incident heart failure in black and non‐black men → HR = 0.66 (95% CI, 0.51–0.85)^(^ ^10^ ^)^	Incident CV disease increased the risk of vertebral fractures compared to disease free → HR = 1.47 (95% CI, 1.19–1.81)^(^ ^11^ ^)^

Adapted from Rodriguez and colleagues.^(^ ^5^ ^)^

MI = myocardial infarction.

## Why Is It Important to Understand the CV Benefits and Harms of Osteoporosis Medications?

Given this context, there is a natural case suggesting that perhaps improvements in skeletal health and fracture reduction may lead to nonskeletal benefit, particularly where CV diseases are concerned. Indeed, greater appreciation of the need for CV disease management alongside fracture risk is warranted across clinical specialties in order to do our best to improve the overall outcomes experienced by patients. By understanding these nonskeletal benefits and harms, we are doing our due diligence by the patient because treatment uptake is affected markedly by perceived risks of harmful albeit extremely rare side effects; most recently regarding prescription of bisphosphonates (BPs).^(^ ^12^ ^)^ Understanding what treatments can accomplish, in the widest possible sense, may impact the overall cost utility landscape of such medications rather than a narrow focus on bone health alone.

The scientific associations between the skeleton and heart and vessels are strong but has not been translated into a clinical message that bone protection may offer CV protection. For example, in women who experience bone loss over time, increases in systolic blood pressure were greatest in those who experienced the greater reduction in bone density.^(^ ^13^ ^)^ This observation is not limited to women; data from the United States show that in men (and women), CV disease is exhibited by those who experience bone loss.^(^ ^14^ ^)^ We have also begun to appreciate the *vascular‐bone* relationship, of how vascular disease can lead to poorer skeletal outcome. In older women, atherosclerotic vascular disease (ASVD) including vascular calcification is highly prevalent and is associated with a higher risk of fractures.^(^ ^15^ ^)^ Though there is little awareness of this in daily clinical practice, patients with underlying stroke or ischemic heart disease go on to experience near double the number of fractures compared to those who are disease‐free.^(^ ^9^, ^16^ ^)^

Finally, there is a regulatory rationale to understand the CV effects of osteoporosis medications. The latest drugs off the pipeline have been subjected to intense CV scrutiny, which is of course appropriate given their prospective widespread use in an older population. Indeed, the cathepsin K monoclonal antibody, odanacatib, was discontinued by the sponsor (Merck) owing to concerns over increases rates of stroke compared to placebo (1.7% [136/8043] versus 1.3% [104/8028]; hazard ratio [HR] = 1.32; 95% confidence interval [CI], 1.02 to 1.70)^(^ ^17^ ^)^ Furthermore, another monoclonal antibody, against sclerostin, romosozumab, has been approved by the US Food and Drug Administration (FDA) with a black‐box warning for potential increases in stroke and myocardial infarction owing to an imbalance in adjudicated CV events in a large active‐comparator trial.^(^ ^18^ ^)^ In other words, both the patients and the prescriber will need to agree that any short‐term increase in cardiovascular morbidity is more than offset by long‐term prevention of serious fractures. This renewed interest in “off‐target” effects, particularly CV effects, of antifracture medications thus warrants further exploration. This concept is already applied in the diabetes setting where new glucose‐lowering medications must also have satisfactory results for both safety and efficacy in a CV outcome trial.

## What Is the Purpose and Scope of this Review?

The purpose of this review is to provide a comprehensive overview of the antifracture medication prescribing landscape and examine the evidence for CV safety and harms of these medications. This review focuses on recent larger randomized controlled trials (RCTs) with reference to key historical and seminal trials in the development of some medications. Particular attention will be paid to studies conducted in postmenopausal women and older men with osteoporosis/low BMD and to nonhormonal treatments with proven antifracture efficacy. Further, this review will focus on the meaningful collective outcome of the disease processes leading to CV events as properly examining mechanistic pathways such as vascular calcification is challenging in the setting of large RCTs. A previous review on this topic was framed as practical guide for the new clinician.^(^ ^19^ ^)^ This review is also aimed at the prescribing clinician with the additional intention to provide a foundation from which conversations about medication safety can be had with patients. The CV safety of antifracture medications needs to be explored in the context of patients with osteoporosis/low BMD having a high underlying vascular disease burden and so CV outcomes of osteoporosis RCTs may be confounded by indication. Furthermore, this review is also aimed at the interested scientist, as we explore knowledge gaps in the bone‐CV field.

## What Is “Accepted” to Be Known?

### Mixed effects of BPs

#### No apparent mortality benefits

BPs are pyrophosphate analogues that have high affinity for hydroxyapatite crystals in bone with a half‐life of several years. BPs have a long history, with the first clinical use noted in 1969.^(^ ^20^ ^)^ Their main effects appear to be on inhibiting resorption by being taken up by osteoclasts and promoting their apoptosis, thereby tipping the remodeling balance in favor of greater deposition to resorption. The idea that BPs could have some potential CV effects stemmed from observations that etidronate (a first‐generation, non–nitrogen‐containing‐BP) could delay the calcification process in the vasculature and outside the skeleton.^(^ ^21^, ^22^ ^)^ Later generation, nitrogen‐containing BPs including oral alendronate, risedronate, and intravenous (iv) zoledronic acid have been shown to inhibit the farnesyl pyrophosphate synthase pathway in osteoclasts, which is a downstream step from the actions of hydroxymethylglutaryl–coenzyme A (HMG‐CoA) reductase inhibitors (statins) in the production of mevalonate in the cholesterol synthesis pathway.^(^ ^23^ ^)^ Given the robust literature surrounding the cardioprotective and mortality benefits of inhibiting this pathway, BPs too have been studied in this respect with mixed effects.

First, BPs appear to offer no mortality benefit as a class in women and men.^(^ ^24^ ^)^ In a meta‐analysis of 21 trials of BPs (*n* = 22,623 treated) versus placebo (*n* = 20,244), BP treatment was associated with a nonsignificant 5% reduced relative risk (RR) of overall mortality (event rates: 3.78% versus 4.35%; 95% CI for RR, 0.86 to 1.04).^(^ ^24^ ^)^ This finding was replicated in sensitivity analysis of zoledronic acid although with a larger effect size of 22% (4.17% versus 4.07%; RR = 0.88; 95% CI, 0.68 to 1.13). Analysis of alendronate‐only studies (four trials) demonstrated a null effect (1.55% versus 1.61%; RR = 1.00; 95% CI, 0.71 to 1.40). Interestingly, analysis of nitrogen‐containing BPs (essentially combining alendronate, risedronate, and zoledronic acid) trended toward significance (2.98% versus 3.62%; RR = 0.90; 95% CI, 0.81 to 1.00). Furthermore, excluding zoledronic acid from analysis of nitrogen‐containing BPs marginally altered the effect estimate (2.34% versus 2.90%; RR = 0.92; 95% CI, 0.79 to 1.07) suggestive of an oral BP–specific effect. These rates and effect estimates are mentioned in detail as the moderate to large effect sizes and upper limits of the confidence bounds (1.00, 1.04, and 1.07) suggests that a clinically meaningful reduction in mortality cannot be excluded given the weight of evidence and results from observational cohorts. The meta‐analysis did not perform population specific sensitivity analyses (men‐only, hip fracture only, etc.) and thus these data indicate there may yet exist a certain population with osteoporosis who may experience a mortality benefit despite not necessarily being prescribed for this effect. Given the high CV burden in patients with osteoporosis, they may be interested in understanding these potential off‐target effects despite clinical suspicion that they are “too good to be true”.^(^ ^25^ ^)^ Finally, there appeared to be no mortality benefit for BPs in trials longer than 3 years, which was an interesting concept given the perceived risk reduction seen in the Active‐Controlled Fracture Study in Postmenopausal Women With Osteoporosis at High Risk (ARCH) trial occurred in the first 12 months^(^ ^26^ ^)^ and risk reductions appeared evident at approximately the 16‐month mark of the Health Outcomes and Reduced Incidence with Zoledronic Acid Once Yearly–Recurrent Fracture Trial (HORIZON‐RFT) trial by Lyles and colleagues.^(^ ^27^ ^)^ This would point to some transient effect that could potentially be related to medication adherence (observational data support this^(^ ^28^ ^)^) or represent a true, underappreciated clinical effect mediated through the skeleton. There is a complete paucity of head‐to‐head studies examining this and there is limited data exploring these effects in other agents^(^ ^29^, ^30^ ^)^ to elucidate if any potential mortality benefit is mediated through skeletal effects.

#### Zoledronic acid may increase the risk of atrial fibrillation

Despite no apparent effect of BPs as a class on overall mortality, cause‐specific effects have been explored in greater detail. One of the most well‐described effects has been the apparent increase in atrial fibrillation associated with iv zoledronic acid (Table 2). This was first described in the HORIZON Pivotal Fracture Trial by Black and colleagues from 2007.^(^ ^31^ ^)^ This trial followed on from the clinical development of alendronate as an antifracture agent a decade earlier.^(^ ^37^ ^)^ The aim of the HORIZON study was to investigate if the more potent zoledronic acid had similar effects to oral alendronate. The intravenous nature of zoledronic acid was appealing to patients because it was required only once yearly rather than daily or weekly as per the oral formulation, which caused gastric problems. HORIZON enrolled over 7700 women aged between 65 and 89 years and randomly assigned to either 5 mg iv zoledronic acid or placebo both with additional daily calcium (1000 to 1500 mg) and vitamin D (400 to 1200 IU). There was an imbalance in overall adverse events, mostly attributable to postinfusion symptoms. Importantly, this seminal trial provided the first robust description for effects of BPs on atrial fibrillation. In those receiving iv zoledronic acid, there were more atrial fibrillation events (94/3862 [2.4%] versus 73/3852 [1.9%]; RR = 1.28; 95% CI, 0.94 to 1.73) that did not reach statistical significance but was signal enough to prompt evaluation of serious atrial fibrillation. In this instance, the imbalance reached statistical significance (50/3862 [1.3%] versus 20/3852 [0.5%]; RR = 2.49; 95% CI, 1.48 to 4.18). This finding motivated an FDA review of oral alendronate that showed 47 serious atrial fibrillation adverse events (1.5%) in alendronate‐treated and 31 (1.0%) in placebo‐treated during an average of 4 years (HR = 1.51; 95% CI, 0.97 to 2.40), a finding that cannot rule out a clinically important effect. Reassuringly, there was no increased risk of all atrial fibrillation adverse events (81 events [2.5%] versus 71 events [2.2%]; HR = 1.14; 95% CI, 0.83 to 1.57).^(^ ^38^ ^)^ The rationale for why BPs may provoke arrhythmia is uncertain. Some preclinical studies point to potential disruption of calcium handling dynamics in cardiomyocytes by BPs.^(^ ^39^ ^)^ Other important triggers initiating atrial fibrillation may arise from focally discharging cells located most commonly at the pulmonary vein ostia. These foci may lead to frequent atrial ectopy and paroxysms of atrial fibrillation. This effect is particularly relevant to the iv administration, which is clouded by flu‐like symptoms postinjection.^(^ ^40^ ^)^ BPs can also stimulate release of inflammatory cytokines, which are implicated in increasing the risk of atrial fibrillation but through mechanisms that have not been fully elucidated.^(^ ^41^, ^42^ ^)^ These data were enough to include in atrial fibrillation as a rare but of uncertain causal origin side effect that clinicians should be aware of in current regulatory agency recommendations.^(^ ^43^ ^)^ More recent data cast doubt on this understanding. A recent trial of similar design to the HORIZON trial specifically enrolled 2000 women in the osteopenic range (that is, having a BMD *T*‐score between −2.5 and −1.0). In a secondary publication of prespecified safety data, Reid and colleagues^(^ ^32^, ^33^ ^)^ demonstrated similar event rates for any atrial fibrillation 14.8 (95% CI, 11.9 to 18.3) events per 1000 zoledronic acid treated and 15.6 (95% CI, 12.6 to 19.1) events per 1000 placebo‐treated women. The crude event rates were much larger than HORIZON for both all atrial fibrillation events (8.8% versus 9.5%; RR = 0.95; 95% CI, 0.72 to 1.26) and for number of women with at least one episode of atrial fibrillation (5.4% versus 5.5%; RR = 0.98; 95% CI, 0.68 to 1.41). In comparing HORIZON with the more recent trial in patients with osteopenia, it may be argued that the women with osteopenia have a lesser underlying CV disease burden (given the shared underlying risk factors for both CV disease and osteoporosis), and thus the administration of zoledronic acid was not sufficient to provoke paroxysms. Cohort studies have demonstrated inconsistent findings regarding this pre‐presumptive association and thus continual investigation and post‐market monitoring is prudent.^(^ ^44^ ^)^

**Table 2:** Cardiovascular Outcomes in Example Major Bisphosphonate RCTs and Meta‐Analyses

Outcome	Trial	Approach	Bisphosphonate *n*/*N* (%)	Comparator *n*/*N* (%)	RR (95% CI)
Atrial fibrillation	Black and colleagues ^(^ ^31^ ^)^ HORIZON (2007)	Zoledronic acid versus placebo	94/3862 (2.4)	73/3852 (1.9)	1.28 (0.94–1.73)
	Reid and colleagues ^(^ 32, 33 ^)^ (2020, 2018)	Zoledronic acid versus placebo	54/1000 (5.4)	55/1000 (5.5)	0.98 (0.68–1.41)
	Cummings and colleagues ^(^ ^34^ ^)^ (1998)	Alendronate versus placebo	82/3236 (2.5)	71/3223 (2.2)	1.15 (0.84–1.57)
Myocardial infarction	Black and colleagues ^(^ ^31^ ^)^ HORIZON (2007)	Zoledronic acid versus placebo	38/3862 (0.9)	45/3852 (1.1)	0.84 (0.54–1.29)
	Reid and colleagues ^(^ 32, 33 ^)^ (2020, 2018)	Zoledronic acid versus placebo	39/1000 (3.9)	43/1000 (4.3)	0.58 (0.35–0.94)
	Kim and colleagues ^(^ ^35^ ^)^ (2016)	Meta‐analysis	69/6154 (1.1)	68/5906 (1.1)	0.96 (0.69–1.34)
Stroke	Black and colleagues ^(^ ^31^ ^)^ HORIZON (2007)	Zoledronic acid versus placebo	87/3862 (2.2)	88/3852 (2.2)	0.98 (0.73–1.32)
	Reid and colleagues ^(^ 32, 33 ^)^ (2020, 2018)	Zoledronic acid versus placebo	20/1000 (2.0)	22/1000 (2.2)	0.90 (0.49–1.66)
	Kim and colleagues ^(^ ^35^ ^)^ (2016)	Meta‐analysis	275/15152 (1.8)	204/10059 (2.0)	0.99 (0.82–1.19)
Any CAE or MACE	Black and colleagues ^(^ ^31^ ^)^ HORIZON (2007)	Zoledronic acid versus placebo	184/3862 (4.7)	177/3852 (4.5)	1.01 (0.84–1.26)
	Reid and colleagues ^(^ 32, 33 ^)^ (2020, 2018)	Zoledronic acid versus placebo	71/1000 (7.1)	98/1000 (9.8)	0.72 (0.54–0.97)
	Kranenburg and colleagues ^(^ ^36^ ^)^ (2016)	Meta‐analysis	1054/12582 (8.3)	678/9338 (7.2)	1.03 (0.91–1.17)

CAE = cardiovascular adverse event.

#### Zoledronic acid may reduce the risk of myocardial infarction

Contrary to the history of zoledronic acid and atrial fibrillation, rates of myocardial infarction have not been shown to be increased with zoledronic acid treatment. In the abovementioned trial by Reid and colleagues,^(^ ^32^, ^33^ ^)^ osteopenic women experienced no increased rate of myocardial infarction, and indeed may even have experienced a reduced rate of infarcts (39 [3.9%] versus 43 [4.3%]; RR = 0.58; 95% CI, 0.35 to 0.94). Survival analysis demonstrated a potential 40% improved survival in zoledronic acid–treated women (HR = 0.60; 95% CI, 0.36 to 1.00) was shown, where the survival curves began to deviate at approximately 18 months.^(^ ^32^, ^33^ ^)^ These data, although prespecified, were secondary endpoints and thus warrant interpretation with caution. This effect is another point of difference between the trial by Black and colleagues^(^ ^31^ ^)^ in women with osteoporosis. There were fewer myocardial infarction events in zoledronic acid–treated women (38 [1.0%] versus 45 [1.2%]), but this did not reach statistical significance (RR = 0.84; 95% CI, 0.54 to 1.29). Similar to effects on overall mortality, these data may indicate that BP may exhibit some transient effect which is support by cohort data on medication adherence. Furthermore, given the potential action of BP on the cholesterol synthesis pathway, there is biological rationale to support an effect on ASVD. Further data are needed to understand if the background ASVD is important in determining CV effects on BPs or if indeed BPs have clinically significant effects in reversing ASVD as per statins. Abdominal aortic calcification (AAC) is recognized to be a surrogate indicator of ASVD and indeed predicts CV and non‐CV outcomes.^(^ ^15^, ^45^, ^46^, ^47^ ^)^ In an exploratory substudy of the HORIZON‐PFT, progression of AAC was examined following 3 years of treatment. AAC progression (defined as a change in semiquantitative AAC score) was similar between treatment groups (67/234 [28.6%] in zoledronic acid–treated versus 82/268 [30.6%] in placebo‐treated; odds ratio [OR] = 0.90; 95% CI, 0.6 to 1.3).^(^ ^48^ ^)^ This did not differ by baseline AAC score, nor did change in total hip (*r* = −0.02, *p* = 0.66) or femoral neck BMD (*r* = 0.03, *p* = 0.54) correlate with AAC change. This overall suggests against a link between the skeleton and calcification in the vasculature as a potential mechanism explaining CV benefits or harms of BPs. However, it should be noted that the semiquantitative AAC scale (0 to 8) may not be sensitive enough to detect changes over the follow‐up period and the intraobserver fidelity was moderate (0.62). Studies using more sensitive techniques and scoring systems may yet observe differences. Understanding subtle changes in AAC is important as it remains to be seen if an AAC score of 0 has similar prognostic power as a coronary artery calcium score of 0.^(^ ^49^, ^50^ ^)^

#### Oral bisphosphonates have a good safety profile regarding CV events

Similar to the seminal trials by Black and colleagues^(^ ^37^ ^)^ and Liberman and colleagues^(^ ^51^ ^)^ in the clinical development of alendronate, trials of other oral formulations have shown similar CV effects. In a trial of 1200 postmenopausal women younger than 85 years; oral risedronate either 2.5 mg/day or 5.0 mg/day did not increase the rate of CV events. Compared to placebo (38 events [9.3%]), there were fewer events in women treated with 2.5 mg (30 [7.4%]; RR = 0.78; 95% CI, 0.49 to 1.24) and the same event rate in women treated with 5.0 mg (38 [9.3%]; RR = 1.00; 95% CI, 0.65 to 1.24). When compared head‐to‐head there was a nonsignificant increased rate of events in those treated with the higher dose (RR = 1.27; 95% CI, 0.80 to 2.01), which suggests against a dose‐response effect.^(^ ^52^ ^)^

#### Denosumab has null effects on CV events

Denosumab is a fully human monoclonal antibody against the receptor activator of nuclear factor‐κB ligand (RANKL), which exerts potent antiresorptive effects. The largest trial conducted in the clinical development of denosumab was the Fracture Reduction Evaluation of Denosumab in Osteoporosis Every 6 Months (FREEDOM) study.^(^ ^30^ ^)^ This double‐blind, placebo‐controlled trial enrolled 7808 postmenopausal women aged 60 to 90 years. Prespecified safety data included serious CV events, strokes, and coronary heart disease. Analysis of these secondary endpoints demonstrated no statistical difference in event rates between the treatment groups (Table 3). For all serious CV events (186 [4.8%] versus 178 [4.6%]; RR = 1.04; 95% CI, 0.85 to 1.27) and strokes (56 [1.4%] versus 54 [1.4%]; RR = 1.03; 95% CI, 0.71 to 1.49) there was a balance in event rates; whereas there was a nonstatistically significant reduced event rate in terms of coronary heart disease (47 [1.2%] versus 39 [1.0%]; RR = 0.87; 95% CI, 0.59 to 1.28). These data are supported by a series of meta‐analyses.^(^ ^55^, ^56^, ^57^ ^)^ Similar to the HORIZON substudy on AAC, RANKL inhibition appeared to not change the fate of AAC progression in a substudy of the FREEDOM trial (118/544 [22%] in denosumab versus 109/501 [22%] in placebo). This finding was consistent across baseline AAC status (score ≤6 versus score >6) and, importantly, renal function (estimated glomerular filtration rate ≤45 versus >45).^(^ ^58^ ^)^ Again, this supports a view that a mechanism independent of calcium handling may explain the benefits or harms of antiresorptive therapies.

**Table 3:** Cardiovascular Outcomes in Example Major Denosumab RCTs and Meta‐Analyses

Outcome	Trial	Comparison	Denosumab *n*/*N* (%)	Comparator *n*/*N* (%)	RR (95% CI)
Atrial fibrillation	Cummings and colleagues ^(^ ^30^ ^)^ FREEDOM (2009)	Placebo	29/3886 (0.7)	29/3876 (0.7)	0.99 (0.59–1.66)
	Brown and colleagues ^(^ ^53^ ^)^ (2009)	Alendronate	1/593 (0.1)	0/586 (0.0)	n/e
Myocardial infarction	Cummings and colleagues ^(^ ^30^ ^)^ FREEDOM (2009)	Placebo	47/3886 (1.2)	39/3876 (1.0)	0.87 (0.59–1.28)
	Brown and colleagues ^(^ ^53^ ^)^ (2009)	Alendronate	1/593 (0.1)	3/586 (0.5)	0.32 (0.03–3.15)
Stroke	Cummings and colleagues ^(^ ^30^ ^)^ FREEDOM (2009)	Placebo	56/3886 (1.4)	54/3876 (1.3)	1.03 (0.71–1.49)
	Brown and colleagues ^(^ ^53^ ^)^ (2009)	Alendronate	1/593 (0.1)	3/586 (0.5)	0.32 (0.03–3.15)
Any CAE or MACE	Cummings and colleagues ^(^ ^30^ ^)^ FREEDOM (2009)	Placebo	186/3886 (4.8)	178/3876 (4.6)	1.04 (0.85–1.27)
	Roux and colleagues ^(^ ^54^ ^)^ (2014)	Risedronate	6/429 (1.3)	4/429 (1.0)	1.52 (0.43–5.37)
	Seeto and colleagues ^(^ ^55^ ^)^ (2021)	Placebo	439/4725 (9.3)	399/4467 (8.9)	1.09 (0.95–1.23)
	Seeto and colleagues ^(^ ^55^ ^)^ (2021)	Bisphosphonate	85/2136 (3.9)	58/2131 (2.7)	1.46 (1.05–2.02)

CAE = cardiovascular adverse event; n/e = not estimable.

#### Parathyroid hormone analogues have null effects on CV events

Parathyroid hormone (PTH) stimulates bone resorption physiologically in order to maintain appropriate circulating calcium levels. However, the osteoclasts responsible for bone resorption release chemokines and other hormones that eventually promote the maturation of osteoblasts resulting in increases in bone formation. This apparent paradoxical effect is utilized clinically, where synthetic analogues of PTH such teriparatide (PTH 1–34, the first 34 amino acids of the hormone) and abaloparatide (PTH‐related protein analogue) are the chief anabolic agents in the physician's armamentarium. In terms of CV safety, the key trial in the clinical development of teriparatide by Neer and colleagues in 2001,^(^ ^59^ ^)^ reported a near balance of cardiac arrhythmias (9/1082 [0.8%] teriparatide‐treated versus 12/1088 [1.1%] placebo‐treated; RR = 0.75; 95% CI, 0.31 to 1.78) and no reports of CV mortality, strokes, ischemic events, or other CV‐related endpoints.^(^ ^59^ ^)^ Abaloparatide is a peptide that selectively binds to the RG conformation of the PTH type‐1 receptor and is hypothesized to have greater skeletal effects than teriparatide. In the pivotal trial for its clinical development over 2400 postmenopausal women were randomized to either abaloparatide, teriparatide, or placebo for 18 months. In the safety analysis, there were similar events rates compared to placebo for the endpoints of myocardial infarction (1/822 [0.12%] abaloparatide‐treated versus 2/820 [0.24%] placebo‐treated]; stroke (3/820 [0.37%] versus 0/822 [0.00%]) and atrial fibrillation (0/820 [0.00%] versus 0/822 [0.00%]), which was similar to the experience with teriparatide two decades earlier.^(^ ^60^ ^)^ Interestingly, in the majority of trials, the direction of effect supports a notion of CV risk reduction, which indirectly supports the underlying theory of skeletal health being linked to CV health (Table 4).

**Table 4:** Cardiovascular Outcomes in Example Major PTH‐Analogues RCTs

Outcome	Trial	Approach	PTH‐analogue *n*/*N* (%)	Comparator *n*/*N* (%)	RR (95% CI)
Atrial fibrillation	Geusens and colleagues ^(^ ^61^ ^)^ (2018)	Teriparatide versus risedronate	3/683 (0.4)	5/683 (0.7)	0.60 (0.14–2.50)
	Miller and colleagues ^(^ ^60^ ^)^ (2016)	Abaloparatide & teriparatide versus placebo	0/1640 (0.0)	0/820 (0.0)	n/e
Myocardial infarction	Geusens and colleagues ^(^ ^61^ ^)^ (2018)	Teriparatide versus risedronate	3/683 (0.4)	5/683 (0.7)	0.60 (0.14–2.50)
	Miller and colleagues ^(^ ^60^ ^)^ (2016)	Abaloparatide & teriparatide versus placebo	3/1640 (0.2)	3/820 (0.3)	0.50 (0.10–2.47)
Stroke	Geusens and colleagues ^(^ ^61^ ^)^ (2018)	Teriparatide versus risedronate	6/683 (1.0)	6/683 (1.0)	1.00 (0.32–3.08)
	Miller and colleagues ^(^ ^60^ ^)^ (2016)	Abaloparatide & teriparatide versus placebo	6/1640 (0.3)	6/820 (0.7)	0.50 (0.16–1.54)
Any CAE or MACE	Geusens and colleagues ^(^ ^61^ ^)^ (2018)	Abaloparatide & teriparatide versus placebo	9/1640 (0.5)	10/820(0.1)	0.45 (0.18–1.10)
	Miller and colleagues ^(^ ^60^ ^)^ (2016)	Teriparatide versus risedronate	9/683 (0.1)	11/683 (0.1)	0.81 (0.34–1.96)
	Ferrieres and colleagues ^(^ ^57^ ^)^ (2020)	Meta‐analysis versus placebo	2/1305 (0.1)	3/1312 (0.2)	0.67 (0.11–4.00)

CAE = cardiovascular adverse event; n/e = not estimable.

#### Romosozumab may increase the risk of ischemic events

Romosozumab is a monoclonal antibody that binds and inhibits sclerostin, with a dual effect of increasing bone formation and decreasing bone resorption. In a trial of romosozumab against placebo, the Fracture Study in Postmenopausal Women with Osteoporosis (FRAME) trial (~3600 postmenopausal women each arm), there was no imbalance in the number of adjudicated serious CV events (44 [1.2%] in romosozumab‐treated versus 41 [1.1%] in placebo‐treated (RR = 1.07; 95% CI, 0.70 to 1.63). Similarly, there was no imbalance in the number of adjudicated CV deaths (17 [0.5%] versus 15 [0.5%]; RR = 1.13; 95% CI, 0.56 to 2.26).^(^ ^62^ ^)^ In contrast, in a similar trial of romosozumab against placebo in older men (*n* = 245), the BRIDGE trial, there was a nonstatistically significant imbalance in adjudicated serious CV events (8/163 [4.9%] in romosozumab‐treated versus 2/81 [2.5%] placebo‐treated; RR = 1.98; 95% CI, 0.43 to 9.14). This imbalance appeared to be driven by an increase in cardiac ischemic (3/163 (1.8%) versus 0/81 [0.00%]) and cerebrovascular events (3/163 [1.8%] versus 1/81 [1.2%]).^(^ ^63^ ^)^ This small signal was also evident in the active‐controlled trial in postmenopausal women, the ARCH trial. The comparator in this trial of over 4000 (~2000 in each arm) postmenopausal women was oral alendronate. The number of adjudicated serious CV events was higher in those treated with romosozumab for the first 12 months compared to alendronate (50 [2.5%] versus 38 [1.9%]; RR = 1.29; 95% CI, 0.85 to 1.97). Again, this imbalance seemed to be driven by ischemic events as there were more myocardial infarctions (16 [0.8%] versus 6 [0.3%]; RR = 2.25; 95% CI, 0.93 to 5.47) and strokes (16 [0.8%] versus 7 [0.3%]; RR = 2.25; 95% CI, 0.93 to 5.47), though neither reached statistical significance. There was no imbalance in the number of CV deaths (17 [0.8%] versus 12 [0.6%]; RR = 1.39; 95% CI, 0.66 to 2.92).^(^ ^18^ ^)^ Of note, participants in the ARCH trial included the highest proportion of patients with a previous history of CV disease 73%, compared to 66% and 65%, in Placebo‐Controlled Study Evaluating the Efficacy and Safety of Romosozumab in Treating Men With Osteoporosis (BRIDGE) and FRAME, respectively. Thus, it has been suggested that perhaps the drug treatment has exacerbated (or provoked) already underlying CV disease and thus a black box warning for romosozumab is in place for any patient who has experienced a major CV event.^(^ ^64^, ^65^, ^66^ ^)^ This position is not supported by recent a meta‐analysis, where romosozumab was not shown to increase the risk of a composite cardiovascular outcome of stroke, coronary artery disease, heart failure, and atrial fibrillation (RR = 1.26; 95% CI, 0.95 to 1.68) or a three‐point major adverse cardiovascular event (MACE) outcome (RR = 1.41; 95% CI, 0.99 to 2.02). However, the lower bounds of the CIs in these estimates cannot exclude a statistically or clinically important effect, that is to say the lower bounds of the CIs only just cross unity; therefore, the majority of the effect lies in the positive direct (namely more events meaning romosozumab is associated with relative harm). Interestingly, there was an increase in a four‐point MACE outcome (RR = 1.39; 95% CI, 1.01 to 1.90). This suggests that assessing CV risks of romosozumab is limited by power (given that a four‐point outcome includes more events than a three‐point outcome). Therefore an event‐driven analysis and ongoing postmarketing analysis should be considered.^(^ ^56^ ^)^

## Emerging topics in the cardiovascular effects of antifracture medications

### Differential arrhythmic and atherogenic effects of oral and intravenous bisphosphonates?

Meta‐analyses have proven largely unhelpful in clarifying the potential harmful and/or beneficial effects of bisphosphonates on cardiovascular outcomes.^(^ ^35^, ^36^, ^67^, ^68^, ^69^ ^)^ There exists much variation in analysis approach (any versus specific BP), included literature (RCTs, industry sponsored only studies, cohort studies, and case‐control studies) and outcomes. Collectively, the message is unclear. In the case of drugs with extensive experience, such as BPs with over 50 years of clinical use, real‐world evidence can complement RCT evidence.^(^ ^70^ ^)^ There could be several reasons why many RCTs of BPs on CV outcomes have not provided firm conclusions. For example, the trials may be underpowered to detect an adequate number of events, the trial being too short (again a question of being event‐driven) and population characteristics (did the patients have too severe CV disease to benefit from mild risk reduction or too mild a disease so not enough events occur over follow‐up). Recent real‐world evidence for CV effects of BPs appear to indicate cardioprotection and possibly a signal toward differential atherogenic and arrhythmic effects. In a cohort of individuals undergoing bone density testing (clinically matched on indication), oral BP (96% alendronate) use was associated with a reduction in atrial fibrillation, heart failure, and stroke but not myocardial infarction.^(^ ^71^ ^)^ Small experimental studies in humans have emerged attempting to define the mechanism for this demonstrating acute QT effects on electrocardiography (ECG) following zoledronic acid infusion.^(^ ^72^ ^)^ However, this may just be a feature of the autonomic response to an infusion. Further to differential atherogenic and arrhythmic effects, other cohort studies indicate there may be differential oral and iv effects of BPs, with the iv zoledronic acid associated with greater harms than oral BPs.^(^ ^44^ ^)^ This is supported by a meta‐analysis of the pivotal RCTs for ibandronate, which showed that although ibandronate was deemed to be safe overall, there was a greater crude rate of atrial fibrillation events in patients receiving intravenous ibandronate (0.5%) compared to the oral formulation (0.3%).^(^ ^73^ ^)^

### Denosumab safety may support the cardioprotective hypothesis of BPs

The context of intense CV scrutiny of romosozumab and BPs was the scientific motivation to reanalyze the specific CV safety data of denosumab. Two previous meta‐analyses have reported on the CV safety of denosumab, but findings were limited, including omission of the pivotal trial in the clinical development of denosumab that reported CV safety.^(^ ^56^ ^)^ In patients with osteoporosis, it was revealed that compared to placebo (an analysis almost entirely dominated by the FREEDOM trial), there was no difference in rates of CV events (RR = 0.79; 95% CI, 0.41 to 1.52). However, when compared to any BP control, there was a 46% increased risk of CV events in denosumab‐treated women (RR = 1.46; 95% CI, 1.05 to 2.02). There was a doubling of risk when considering a five‐point MACE outcome (RR = 2.33; 95% CI, 1.19 to 4.56).^(^ ^55^ ^)^ These findings mirror what was evidenced by the romosozumab trials—namely treatment arms experiencing elevated CV events in a BP‐controlled trial but not in a placebo‐controlled trial. This provides indirect evidence for cardioprotection by BPs. Furthermore, recent studies in postmenopausal women suggest that RANKL inhibition does not alter markers of ASVD, further supportive of the imbalance of events seen in the meta‐analysis being attributable to suppression of events in BP arms.^(^ ^74^ ^)^ Of note, the meta‐analysis did not perform sensitivity analyses for BP type to elucidate if effects were specific to oral or iv formulations, so direct comparisons to the zoledronic acid trials are limited. This is particularly relevant given that the MACE outcome only reached significance upon inclusion of atrial fibrillation an outcome for which zoledronic acid has perceived effects.

### PTH‐analogues may have autonomic heart rate effects of clinical importance

At the request of the FDA, investigators of the “Abaloparatide Comparator Trial in Vertebral Endpoints” (ACTIVE) study performed a comprehensive post hoc cardiovascular safety study of trial participants.^(^ ^75^ ^)^ Heart rates, blood pressure, and adverse events presumed related to increased heart rates were recorded. Overall rates of treatment‐emergent adverse events were higher in participants receiving study medications as expected. However, treatment‐emergent adverse events related to elevated heart rates that lead to trial discontinuation were substantially more frequent in abaloparatide‐treated (27/822; 3.3%) and teriparatide‐treated (11/818; 1.3%) than placebo (5/820; 0.6%). By contrast, unadjudicated MACE was approximately half as frequent in abaloparatide (4/822; 0.5%) and teriparatide (5/818; 0.6%) compared to placebo (14/820; 1.7%), representing hazard reductions of 57% (0.43; 95% CI, 0.13 to 1.34) in abaloparatide‐treated and 51% (0.49; 95% CI, 0.17 to 1.44) in teriparatide‐treated. In a long‐term follow‐up to these participants, where all participants went on to receive alendronate from study months 19 to 42, there was no imbalance in events (7/533 [1.3%] versus 7/580 [1.2%]; HR = 0.67; 95% CI, 0.31 to 1.43). These represent the most in‐depth, published data from RCT participants on direct cardiac effects of osteoporosis medications. Although hazard reductions were nonsignificant, given PTH analogues are anabolic, this trial provides indirect evidence to support the theory that bone health has an inverse relationship with cardiac health.

### Ongoing uncertainty of the cardiovascular significance of romosozumab

There has been substantial interest in understanding if sclerostin inhibition imposes CV risk since the publication of the ARCH trial (Table 5). This was recently reviewed briefly by Langdahl and colleagues^(^ ^76^ ^)^ and Fixen and Tunoa.^(^ ^77^ ^)^ Both reviews concluded romosozumab to be safe in this respect and warned against overinterpreting low event rates. There is biological rationale for why this may be the case. In a murine model of atherosclerosis, the SOST gene (which encodes sclerostin) was expressed in the aorta and upregulation of *SOST* conferred vasculoprotection.^(^ ^78^ ^)^ This finding supports the clinical experience of patients with sclerosteosis, a genetic mutation leading to the overexpression of the SOST gene and thus exhibit naturally high levels of sclerostin. These people exhibit marked bone deposition, but importantly, appear to be at no increased risk of CV disease.^(^ ^79^ ^)^ This would seem at odds with a uniquely designed genetic analysis of single nucleotide polymorphisms (SNPs) of the SOST gene associated with increased BMD from several large population databases. This analysis revealed at least one SNP that imposed an elevated CV risk, particularly for myocardial infarction and coronary revascularization.^(^ ^80^ ^)^ This finding would support the black‐box warning by the FDA. However, when considered in the context of myocardial infarction risk reduction in the zoledronate trial of osteopenic women, suggestive of cardioprotection by BPs, these data provide a compelling research justification an RCT with a vascular primary outcome. Presently, it is still uncomfortably uncertain if the romosozumab increases CV events or BPs suppress CV events.

**Table 5:** Cardiovascular Outcomes in the Seminal Romosozumab RCTs

Trial	Romosozumab *n*/*N* (%)	Control *n*/*N* (%)	RR (95% CI)
FRAME ^(^ ^62^ ^)^ (versus placebo) ‐ All	44/3581 (1.2)	41/3575 (1.1)	1.07 (0.70–1.63)
Ischemic events	2 (0.05)	2 (0.05)	0.99 (0.14–7.08)
Cerebrovascular events	8 (0.22)	9 (0.25)	0.88 (0.34–2.29)
ARCH ^(^ ^18^ ^)^ (versus alendronate) ‐ All	50/2040 (2.5)	38/2014 (1.9)	1.29 (0.85–1.97)
Ischemic events	**16 (0.8)**	**6 (0.3)**	**2.63 (1.03–6.71)**
Cerebrovascular events	16 (0.8)	7 (0.3)	2.25 (0.93–5.47)
BRIDGE ^(^ ^63^ ^)^ (versus placebo) ‐ All	8/163 (4.9)	2/81 (2.5)	1.98 (0.43–9.14)
Ischemic events	3 (1.8)	0 (0.0)	n/e
Cerebrovascular events	3 (1.8)	1 (1.2)	1.49 (0.15–14.11)

Bold values signifies statistically significant.

n/e = not estimable.

## The Future

In the big data age, it is becoming easier to investigate in a rapid and high throughout manner, “off‐target” effects of common medications, albeit not without the risk of confounding. There is a business case for this; most drugs have a finite lifespan under patent so the window for pharmaceutical companies to financially exploit their invention is limited. Repurposing of existing medications known to be safe by expanding the indication thus has enormous attraction. A recent success story of this has been the CV efficacy of sodium‐glucose co‐transporter 2 inhibitors in patients without diabetes.^(^ ^81^ ^)^ Equally, from a public health standpoint there is merit in looking for and confirming putative “off‐target” effects. Many osteoporosis medications, particularly alendronate, are currently available as a generic. Thus, if a genuine “off‐target” effect is established (that is to say, demonstrated in an adequately powered RCT) and regulatory authorities grant an expanded indication, this could potentially save healthcare systems a significant amount of money. This is already happening, one example being ticagrelor. Originally marketed as an anti‐platelet agent for the treatment and prevention of stroke and coronary outcomes, the pivotal efficacy trial demonstrated reduced pulmonary events, and this has been followed up with studies demonstrating antimicrobial properties.^(^ ^82^, ^83^, ^84^ ^)^ The natural endpoint of repurposing is eventual incorporation into the approval process. There is precedent for this, as new agents for diabetes must now demonstrate safety as well as CV efficacy. One may argue that the links between bone disease and CV disease is just as strong as that between diabetes and CV disease. Thus, given the risk of CV outcomes imposed by osteoporosis and the high ASVD burden in this population, there can be an argument for this to also occur for new skeletal agents. The experience of romosozumab shows that the field seeks this evidence; and it would be wiser to have this data produced in the setting of a large, well‐designed RCT rather than relying on the eventual churn of reviews and subpar meta‐analyses. Such large, well‐designed RCTs are near prohibitively expensive and pharmaceutical companies are likely to resist subjecting their novel agents to this scrutiny. Thus, there is a role for well‐designed cohort studies to provide real‐world evidence and work in tandem with RCTs.^(^ ^85^, ^86^ ^)^

## Conclusion and Research Agenda

The literature examining the potential CV harms or benefits of antifracture medications is now quite substantial. This is helped by more rigorous reporting, where registered trials are required to report adverse drug reactions (ADRs). The systematic collection of ADRs both in trials and population‐wide health systems through electronic medical records and claims databases means that the body of real‐world evidence is substantial. Generally, these findings have been complimentary to findings in RCTs. However, this has not led to firm conclusions. First, adverse event reporting from RCTs means that the majority of evidence on this topic is secondary and subject to bias and being underpowered. Meta‐analyses, in an attempt to overcome the issue of power, have largely proved unhelpful and are often poorly conducted. By way of example, a meta‐analysis of PTH‐analogues included the pivotal trial by Neer and colleagues.^(^ ^59^ ^)^ This trial was included in summary estimates despite not reporting specific CV events in the primary publication nor are data available on the trial's registration page. Not reporting events does not necessarily mean zero events.

The overarching question of the CV safety for antifracture medications can only definitively be answered in an RCT. There is particular need for an RCT of BPs with a primary vascular endpoint in order to allay criticism that interpretation of safety data needs to be cautious. Further, such an RCT needs to be event‐driven to avoid overinterpretation of low event rates. There may be an ethical concern for conducting such a trial of “repurposing” a medication. Is it justified to apply a medication to examine an “off‐target” effect if proven treatments for those “off‐target” effects already exist? However, patients with osteoporosis have a high ASVD burden and likely other adverse risk markers so the balance of benefit and harm may fall marginally in favor of the trial. Overall, there is need to optimize CV management for improved skeletal outcomes. Furthermore, there is need to better understand the clinical and biological links between skeletal and CV disease because antifracture medications appear to have no effect on aortic calcification (a robust marker of both skeletal and CV risks).^(^ ^48^, ^58^ ^)^ Therefore, exploring other mechanisms such as potential electrophysical effects, antithrombotic effects, effects on autonomic function after iv infusion, or plaque stabilization effects may offer a window into the bone‐vascular axis. In conclusion, the clinical message should be that osteoporosis medications have a very good safety record and that there is a clear research need to study the potential CV benefits that may, perhaps surprisingly, accompany restoration of bone health.^(^ ^71^, ^87^, ^88^ ^)^

## Conflicts of Interest

AJR declares no conflicts of interest. BA declares research contracts with Novartis, Kyowa‐Kirin UK, and UCB with funds paid to the institution, and consulting or speakers fees from Amgen, UCB, Eli‐Lilly, Pharmacosmos, and Kyowa‐Kirin UK.

### Peer Review

The peer review history for this article is available at https://publons.com/publon/10.1002/jbm4.10522.
