# Cardiovascular Safety of Antifracture Medications in Patients With Osteoporosis: A Narrative Review of Evidence From Randomized Studies

**DOI:** 10.1002/jbm4.10628

**Published:** 2022-05-11

**Authors:** Alexander J Rodríguez, Bo Abrahamsen

The authors acknowledge and correct two errors.^(^ ^1^ ^)^ In the Abstract and section titled “Why Is It Important to Understand the CV Benefits and Harms of Osteoporosis Medications?”, we described the anabolic agent odanacatib as a monoclonal antibody. This is incorrect, and it is in fact a chemical compound that acts to inhibit cathepsin K. In the section titled “Ongoing Uncertainty of the Cardiovascular Significance of Romosozumab,” we wrote that in patients with sclerosteosis, SOST gene mutations lead to increased sclerostin levels when in fact it leads to decreased levels. These errors do not affect our overall interpretation of the clinical experience of romosozumab in trials and uncertainty over cardiovascular risk. The authors regret any confusion and inconvenience this may have caused the readers of *JBMR Plus*.
